# Supplementary material for: Liposome Formulation and In Vitro Testing in Non-Physiological Conditions Addressed to Ex Vivo Kidney Perfusion
Source: Int J Mol Sci. 2022 Jul 20;23(14):7999. doi: 10.3390/ijms23147999 (PMC9324182; doi:10.3390/ijms23147999)
Supplement: Supplementary file 1 [file ijms-23-07999-s001.zip › ijms-1829799-supplementary.pdf]

SUPPLEMENTARY DATA

Table S1: dimension, PDI and zeta-potential comparison of liposome's different molar ratio formulation prepared by thin film hydration method followed by extrusion.

Batches	***Molar ratio (DSPC:Chol)***	***Lipids concentration (mM)***	***Dimension (nm ±SD)***	***PDI***	***Zeta-Potential***
A	90:10	8	1916.4±5.66	1.11	+ 1.07
B	70:30	8	1419±4.78	1.73	+ 0.9
C (#1)	50:50	8	163.9±3.25	0.227	+ 1.18
D	30:70	8	252.6±5.90	0.264	+ 1.2
E	10:90	8	251.35±6.00	0.37	+ 1.15
F	50:50	2.7	904.8±82.1	0.49	+ 0.94

Table S2: dimension, PDI and zeta-potential of liposome's batches with different composition and prepared by Nanoassemblr.

Batches	***Molar ratio DSPC:Chol***	***FRR (PBS:Lipids)***	***TFR***	***Dimension (nm ±SD)***	***PDI***	***Zeta-Potential mV***
G	90:10	5:1	8ml/min	132.75±11.52	0.09	+1.3
H	90:10	5:1	12ml/min	140.7±21.21	0.18	+1.14
I	70:30	5:1	8ml/min	265.6±10.08	0.36	+0.96
L	70:30	5:1	12ml/min	213.5±12.3	0.07	+1.0
M	50:50	5:1	8ml/min	113.6±42	0.17	+1.06
N	50:50	5:1	12ml/min	112.9±0.7	0.23	+1.10
O	30:70	5:1	8ml/min	4662.8±3893	0.70	+0.91
P	30:70	5:1	12ml/min	147±47.9	0.70	+0.87
Q	10:90	5:1	8ml/min	384±20.4	0.8	+1.19
R	10:90	5:1	12ml/min	390±13.5	0.85	+1.29
S (#2)	50:50	3:1	8ml/min	112.9±0.7	0.23	+1.11
T	50:50	3:1	12ml/min	124.8±0.56	0.38	+1.07

Table S3 reports the DSPC:Chol liposomes batches obtained using thin film hydration method followed by extrusion at different DSPC:Chol molar ratio and volumes of PBS (pH 7.4) used as rehydrating buffer.

Table S3: Liposome compositions tested by TFH.

Batches	*DSPC (µl)*	*DSPC (mg)*	*DSPC (µmol)*	*Chol(µl)*	*Chol (mg)*	*Chol (µmol)*	*Molar ratio DSPC: Chol*	*Total amount of lipids (µmol)*	*PBS (ml)*
A	800	8	10	43.3	0.43	1.12	90:10	11.24	1.265
B	800	8	10	160	1.6	4.1	70:30	14.1	1.76
**C (#1)**	**460**	**4.7**	**5.9**	**195**	**2.16**	**5.6**	**50:50**	**11.5**	**1.3**
D	197	1.97	2.5	261	2.61	6.75	10:90	9.26	1.16
E	200	2	2.53	654	6.53	16.9	10:90	19.4	0.242
F	460	4.7	5.9	195	2.16	5.6	50:50	11.5	6.433

All liposomes' batches were characterized for their dimension, polydispersity index (PDI), Z-potential and stability (4°C).

Table S2 lists the composition DSPC:Chol liposomes batches obtained using NanoAssemblr technique, variable DSPC:Cholesterol molar ratios, FRR and TFR.

Table S4: Liposome compositions and process parameters tested by microfluidic technique.

Batches	*DSPC:Cholesterol*	*FRR (PBS:Lipid ethanol solution)*	*Lipids volume (ml)*	*Lipids (mg/ml)*	*TFR*
G	90:10	5:1	0.2	0.93	8ml/min
H	90:10	5:1	0.2	0.93	12ml/min
I	70:30	5:1	0.2	1.05	8ml/min
L	70:30	5:1	0.2	1.05	12ml/min
M	50:50	5:1	0.2	1.26	8 ml/min
N	50:50	5:1	0.2	1.26	12 ml/min
O	30:70	5:1	0.2	1.13	8ml/min
P	30:70	5:1	0.2	1.13	12ml/min

Q	10:90	5:1	0.2	1.8	8ml/min
R	10:90	5:1	0.2	1.8	12ml/min
S	**50:50**	**3:1**	**0.25**	**1.58**	**8 ml/min**
T	50:50	3:1	0.25	1.58	12 ml/min

All liposomes' batches were characterized for their dimension, polydispersity index (PDI), Z-potential and stability (4°C).
